# Adipose Weight Gain during Chronic Insulin Treatment of Mice Results from Changes in Lipid Storage without Affecting *De Novo* Synthesis of Palmitate

**DOI:** 10.1371/journal.pone.0076060

**Published:** 2013-09-17

**Authors:** Henriette Frikke-Schmidt, Thomas Åskov Pedersen, Christian Fledelius, Grith Skytte Olsen, Marc Hellerstein

**Affiliations:** 1 Department of Nutritional Sciences and Toxicology, University of California Berkeley, Berkeley, California, United States of America; 2 Department of Insulin Biology, Novo Nordisk A/S, Maaloev, Denmark; 3 Department of Insulin Pharmacology, Novo Nordisk A/S, Maaloev, Denmark; 4 KineMed, Inc., Emeryville, California, United States of America; University College Dublin, Ireland

## Abstract

Insulin treatment is associated with increased adipose mass in both humans and mice. However, the underlying dynamic basis of insulin induced lipid accumulation in adipose tissue remains elusive. To assess this, young female C57BL6/J mice were fed a low fat diet for 3 weeks, treated subsequently with 7 days of constant subcutaneous insulin infusion by osmotic minipumps and compared to mice with only buffer infused. To track changes in lipid deposition during insulin treatment, metabolic labeling was conducted with heavy water for the final 4 days. Blood glucose was significantly lowered within one hour after implantation of insulin loaded mini pumps and remained lower throughout the study. Insulin treated animals gained significantly more weight during treatment and the mean weight of the subcutaneous adipose depots was significantly higher with the highest dose of insulin. Surprisingly, *de novo* palmitate synthesis within the subcutaneous and the gonadal depots was not affected significantly by insulin treatment. In contrast insulin treatment caused accumulation of triglycerides in both depots due to either deposition of newly synthesised triglycerides (subcutaneous depot) or inhibition of lipolysis (gonadal depot).

## Introduction

In addition to lowering of blood glucose, treatment with insulin also induces increases in lipid synthesis and storage [1]. This has implications for insulin treatment of hyperglycemia in diabetic patients, as it has been described in humans as well as rodents that initiation of insulin therapy leads to weight gain that is characterized by an increase in adiposity [2,3].

Several mechanisms exist by which insulin could alter lipid metabolism in liver as well as in white adipose tissues, including: I) transcriptional upregulation of lipogenic enzymes in both types of tissue [4], II) relocation of the GLUT4 transporter to the adipocyte membrane resulting in cellular entrance of glucose enhancing substrate availability [5], III) changing the release of fatty acids from adipose tissues [6], and IV) improving the ability of adipocytes to mature from preadipocytes [7,8].

As adipose tissue mass gain with insulin therapy is considered an adverse effect, it would be an advantage if future insulin therapies could dissociate adipose lipid gain from reduction of hyperglycemia. In order to do so it is crucial to know the interplay between these mechanisms *in vivo*, and to identify the most significant ones. Heavy water labelling is a valuable tool in this setting as it allows for the simultaneous and *in situ* determination of fatty acid synthesis as well as synthesis, storage and release of triglycerides (TGs) in distinct adipose depots [9,10].

In the current study we chose to use an *in vivo* model that was fully insulin responsive to address how hyperinsulinemia affects lipid dynamics in a system that is not insulin resistant or defective. Furthermore, the mice were fed a low fat diet to ensure that *de novo* synthesis of fatty acids was not suppressed by the presence of dietary fat. We found that *de novo synthesis of palmitate* was not induced by insulin treatment under these conditions whereas TG dynamics were altered.

## Methods

### Animals

All animal experiments were approved by the University of California Berkeley Animal Care and Use Committee (Animal Use Protocol # R094). 15 weeks old female C57BL6/J mice (Charles River Breeding Laboratories, Portage, MI) were fed a synthetic low fat diet ad libitum (D10012M, Research Diets, New Brunswick, NJ; 9.4 kcal% fat) for 3 weeks prior to initiation of insulin treatment and throughout the treatment period. The mice were housed in groups of 4 until the day prior to insulin treatment, where they were stratified for weight and blood glucose concentrations and placed in individual cages with ad libitum access to a known amount of food. Right before euthanisation, all food still present in the cage was removed and weighed. The food intake during treatment was calculated as the difference between these two amounts. n=6 within each group.

### Insulin treatment

At the age of 18 weeks the mice had osmotic minipumps (Alzet, Model 1002) inserted subcutaneously under isoflurane anaesthesia (Alzet^®^, Cupertino, CA). The pumps contained either phosphate buffer, 300µM insulin Actrapid^®^ (Low Insulin group “LI”), or 450µM insulin Actrapid^®^ (High Insulin Group “HI”) corresponding to a release of 0 nmol/day, 1.8 nmol/day, and 2.4 nmol/day, respectively (Actrapid^®^, Novo Nordisk, Bagsvaerd, DK). The mice were treated with analgesia using 5mg/kg meloxicam injected intramuscular immediately prior to and for 3 days after impantation (Metacam^®^, Boehringer Ingelheim, Ridgefield, CT). The pumps remained inserted for 7 days, after which the mice were euthanized by cardiac puncture under isoflurane anaesthesia. Throughout the treatment period all mice were weighed and had their blood glucose measured daily (Contour^®^ Blood Glucose Meter, Bayer, Mishawaka, IN).

### ^2^H _2_O labelling protocol

4 days prior to euthanisation each mouse received an IP injection (0.35ml pr g body weight) of 100% ^2^H _2_O with 0.9% NaCl added for isotonicity. The normal drinking water was then replaced with tap water containing 8% ^2^H _2_O. This is known to give a sustained body water enrichment of app. 5% [10].

### Derivatization and analysis of TG-glycerol, palmitate and body water

Upon euthanisation the dorsal subcutaneous and gonadal adipose depots were carefully dissected, weighed, and a piece of each tissue was placed in 2:1 methanol: chloroform for overnight lipid extraction. Body water was retrieved by overnight distillation of whole blood. The lipid extract was processed and prepared for GCMS analysis of palmitate and TG-glycerol as described in detail previously [9]. Body water enrichment was measured as described previously [11].

### Calculations

#### Fractional synthesis

The fraction of newly synthesized TG-glycerol and -palmitate formed during the ^2^H _2_O labeling period was assessed using a combinatorial model of polymerization biosynthesis, as described previously [10,11].

Fractional palmitate synthesis includes all pre-existing, and thus unlabelled, palmitate in the adipose depots, which causes an underestimation of the contribution of de novo palmitate in the newly assembled and deposited TG. To correct for this, the ratio of fractional palmitate synthesis to fractional TG-glycerol synthesis is calculated, representing the fractional contribution of new palmitates within the new TGs only.

#### Absolute rates of palmitate synthesis

The absolute synthesis rate of palmitate in adipose tissue was calculated as fractional synthesis * the weight of the fat pad * 0.8 (the estimated fraction by weight of TG in adipose tissue) * the percentage of FA within the depot that was palmitate. This amount represents the absolute amount (mg) of palmitate synthesised *de novo* during the entire labelling period.

#### TG-glycerol accumulation, synthesis, and lipolysis

In all calculations of the effects of insulin treatment on TG accumulation dynamics, the results are expressed as differences in insulin-treated animals relative to controls rather than changes from pre-treatment values. This was done to avoid the need for estimation of initial fat depot size at the start of treatment. In practice this was done by subtracting the control group mean from all individual values. This results in the difference between the treatment groups (LI and HI) and the control group while preserving the standard variation in the control group for statistical purposes

TG accumulation represents how much more TG mass that was present within each depot at the end of treatment (found by multiplying adipose weight with 0.8, which is the estimated fraction by weight of TG in adipose tissue). Newly formed TG represents the mass of TG that was calculated to be newly synthesised within each depot during treatment. Finally lipolysis is the difference between total accumulated TG and the quantified amount of newly deposited TG (when the actual accumulation of TG is less than what was newly made, this represents the TG that has been escaping via lipolysis). A thorough description of the theory behind these calculations has been described in detail elsewhere [10].

As the treated mice had lower net lipolysis rates than the control group, these difference values became mostly negative.

### Statistical analysis

Statistical significance was tested by one-way or two-way ANOVA as appropriate followed by Bonferroni pairwise comparisons. All data collected from the gonadal depots did not meet the ANOVA requirements for equal variances and were for this reason square root transformed prior to analysis. In those datasets that contained negative numeric values, all numbers had 100 added prior to the transformation for statistical purposes. All data are depicted as mean ± SE except for the data from the gonadal depots where the mean and SE values are back-calculated from the transformed data giving rise to error bars that are not symmetrical. p < 0.05 was considered significant for all tests. All statistical analysis were performed in Graph Pad Prism (version 5, La Jolla, CA)

## Results

### Blood glucose

**Figure 1:**
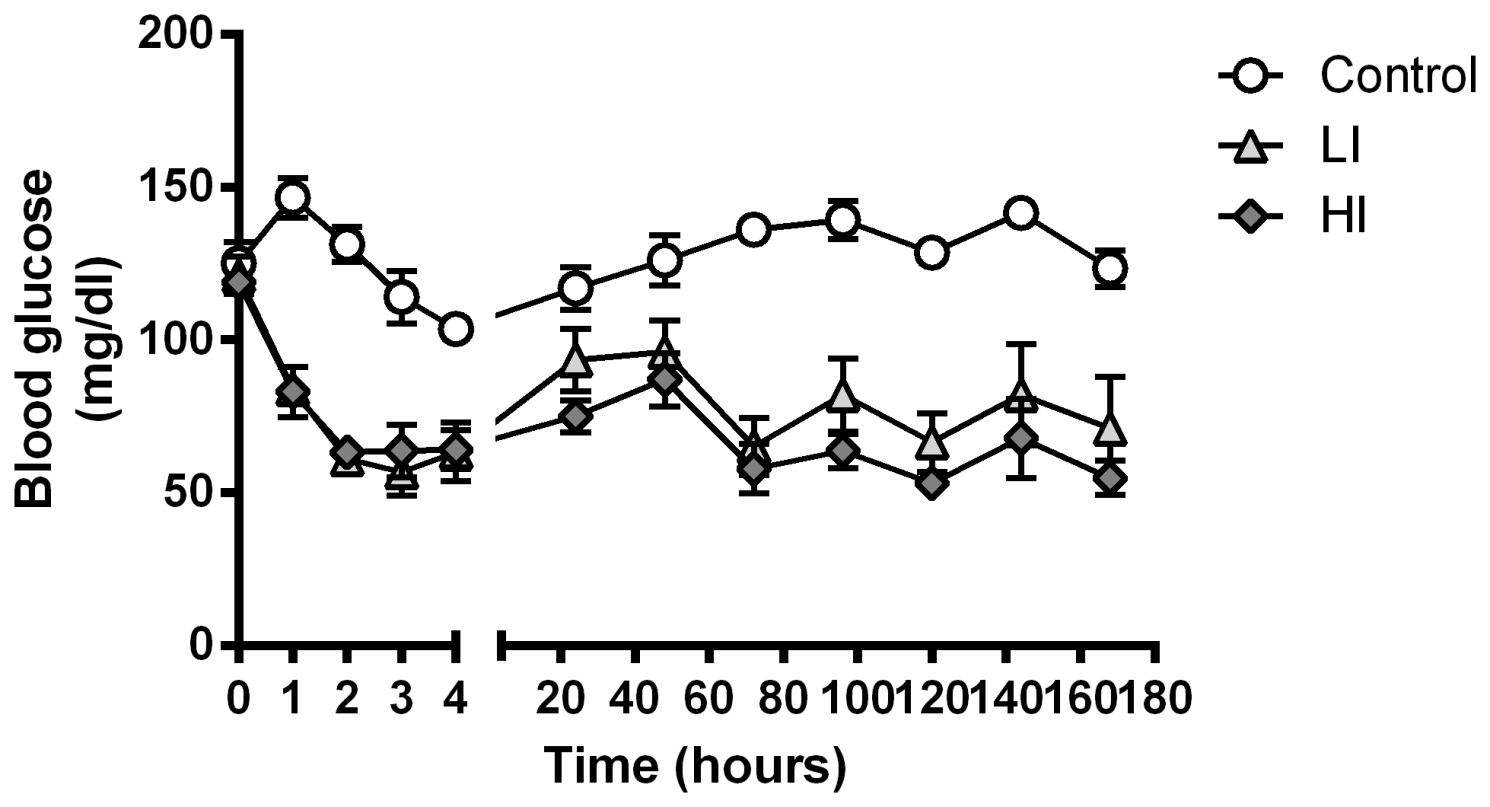
Blood glucose levels. Blood glucose concentrations after subcutaneous implantation of osmotic minipumps containing insulin Actrapid® (Control = 0nmol/day, LI = 1.8nmol7day or HI = 2.4nmol/day, respectively). Error bars indicate SE.

To evaluate the effect of the insulin therapy, the mice had their blood glucose levels measured every day throughout the treatment period. The blood glucose concentrations just prior to pump implantation and during the following 7 days of insulin treatment are depicted in Figure 1. In all insulin treated mice the blood glucose was significantly lowered within the first hour after pump implantation. The blood glucose concentration remained significantly lower throughout the treatment period (p< 0.0001 for all time points for the HI group; p< 0.0001 for 1-3 hours, p < 0.05 at 4 hours, and p < 0.0001 at 72-166 hours for the LI group). The lowest blood glucose measured during the study was 33mg/dl, but no mice showed any physical signs of hypoglycaemia such as tremor, lethargy, or piloerection.

### Body weight and adipose tissue weights

**Figure 2:**
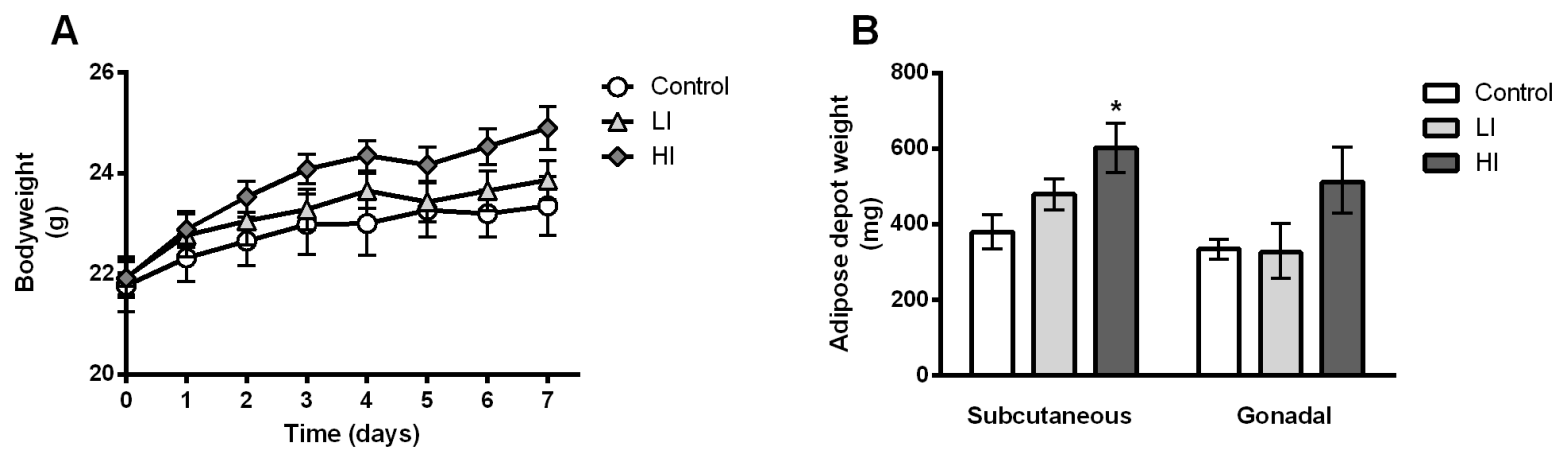
Body weight and adipose depot weights. Body weight after implantation of osmotic minipumps containing insulin Actrapid® (control = 0 nmol/day, LI = 1.8 nmol/day, and HI = 2.4 nmol/day, respectively). B) Weight of the subcutaneous and the gonadal depots, respectively, after 7 days of insulin treatment. * = p < 0.05 as compared to control. Error bars indicate SE.

All mice gained weight during treatment (Figure 2A) and by the end of the treatment the HI group had gained app. 1.5g more than the control group (p < 0.05 for weight gain within HI group as compared to weight gain within the control group).

The weight of the subcutaneous and the gonadal depots, respectively, can be seen in Figure 2B. The weight of the subcutaneous depot in the HI treated animals exceeded the mean weight of the subcutaneous depots in the control group by app. 50% (p < 0.05 for HI vs. control).

### Food intake

The mean food intake during the 7 days of insulin treatment is shown in table 1. There was no statistical difference in food intake between groups, although both the LI and HI groups showed non-significant trends towards higher intake

**Table 1:** Food intake during insulin treatment.

	**Food ingested (g per 7 days**)
**Control**	26.4±3.3
**LI**	28.7±3.8^n.s.^
**HI**	28.7±2.5^n.s.^

Values are expressed as mean ±SD

^n^.^s^. = no significant difference as compared to control

### Fractional and absolute DNL

**Figure 3:**
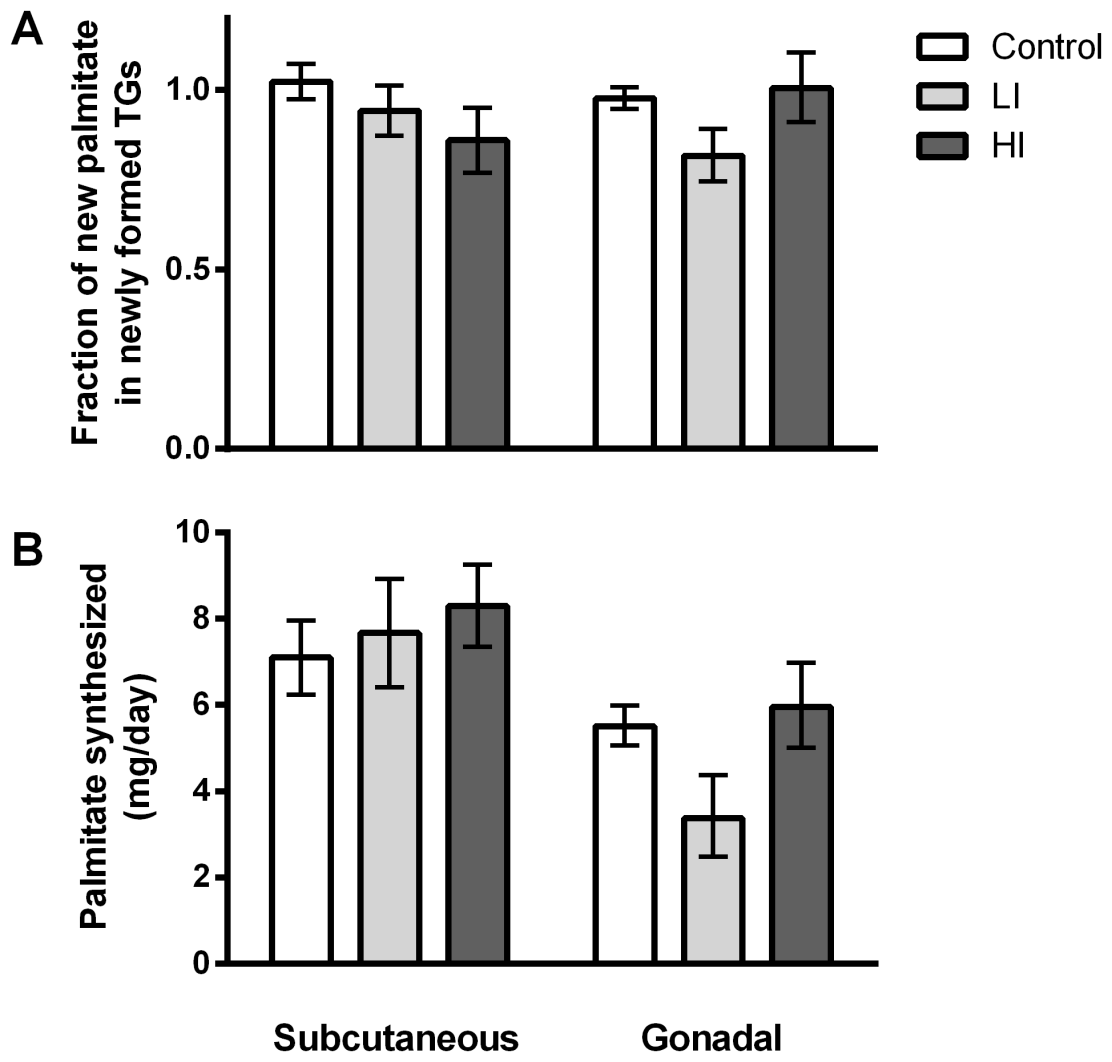
Palmitate synthesis in the adipose depots. Fractional palmitate synthesis contribution to TG-palmitate during the four day labelling period, corrected for turnover of adipose TG. , i.e., an estimate of the fraction of newly synthesized palmitate molecules in new TGs. B) Absolute *de* *novo* palmitate synthesis expressed as mg produced pr day in each fat depot. Error bars indicate SE.

Irrespective of insulin treatment, palmitate incorporated into newly formed TG molecules in adipose tissue depots was mainly *de novo* synthesized (60-80% of newly deposited TG-palmitate derived from the *de novo* synthetic pathway rather than diet or previous TG stores, see Figure 3A) and the absolute amount of newly synthesized palmitate within each depot did not differ significantly between groups (Figure 3B).

### TG-glycerol accumulation, synthesis, and lipolysis

**Figure 4:**
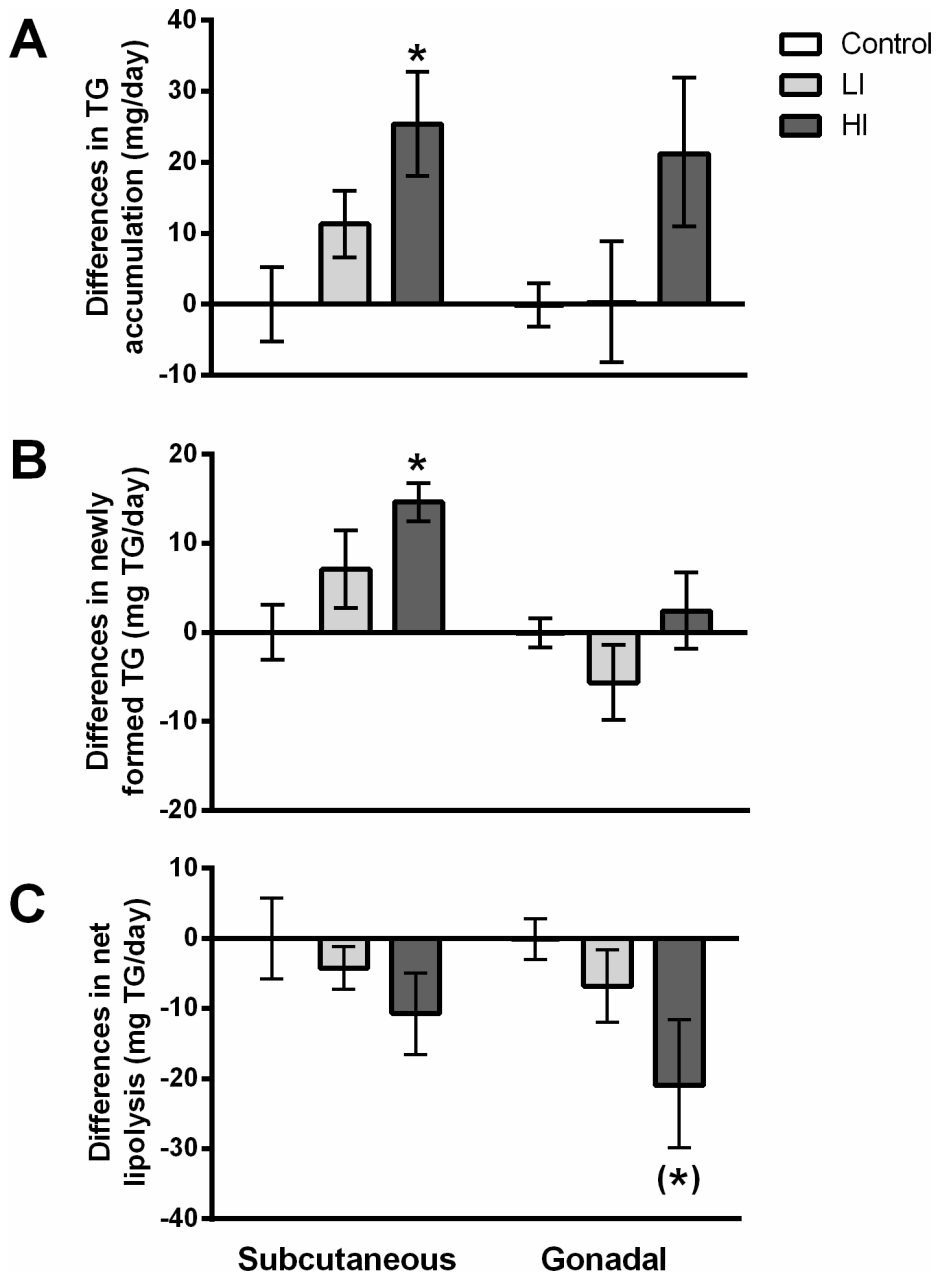
TG dynamics in the adipose depots. TG-glycerol dynamics within the subcutaneous and gonadal depot, respectively. All values are expressed as mg TG/day relative to the control group by subtracting the mean of the control values from all other numbers. A) Difference in TG accumulated pr day in each adipose depot. B) Difference in amount of TG made pr day. C) Difference in amount of TG released from each depot pr day relative to the control group (net lipolysis). * = p < 0.05 as compared to control; ^(^*^)^= p < 0.05 (significance obtained with t-test but was lost in the Bonferroni correction). Error bars indicate SE.

After one week of insulin treatment the HI group had accumulated more TG than the control group (more than 20mg per day in both the subcutaneous and the gonadal depots, respectively), which was found to be statistically significant with the subcutaneous depot (Figure 4A). In the subcutaneous depot most of the TG accumulated in excess of the control group arose from newly synthesized TG molecules (app. 15 mg/day, see Figure 4B) with a smaller and non-significant fraction of this accumulated TG attributable to a decrease in net lipolysis (Figure 4C). In the gonadal depot the opposite was observed: namely, little TG accumulated from newly deposited TG whereas there was a significant difference in excess TG gained relative to the control groups due to inhibition of lipolysis.

## Discussion

In the present study we aimed to elucidate the mechanism behind insulin therapy-induced increases in fat depot mass. We found that the amounts of *de novo* synthesised pamitate within each adipose depot did not change whereas significantly more TG was accumulated due either to increased deposition of new TG molecules (in the subcutaneous depot) or to reduced lipolysis (in the gonadal depot). These effects took place despite a no significant differences in measurable food intake among groups.

The experimental designwas chosen to provide optimal conditions for insulin driven DNL, i.e. a low fat diet was chosen to diminish suppression of *de novo* lipid synthesis due to surplus dietary fat [12] and the animals were fully insulin responsive. Yet we found that insulin therapy induced accumulation of TG without having any effect on the *de novo* synthesis of palmitate, which is the primary product of the DNL pathway from acetyl-CoA. It can be speculated that *de novo* palmiate synthesis might have been negatively affected by the lowered levels of blood glucose resulting in reduced glucose availability in the lipogenic tissues (liver and adipose tissue), giving rise to altered regulation of lipogenic enzymes by e.g. carbohydrate response element binding protein (ChREBP) [13]. Liver and adipose tissues take up glucose by facilitated transport via mainly GLUT2 and GLUT4, respectively [14,15]. However, because the glucose uptake mediated by these transporters is driven by diffusion from a higher concentration towards a lower concentration and as glucose is rapidly converted to glucose-6-phosphate by hexokinase upon entering the intracellular milieu, it is unlikely that less intracellular glucose would be available for fatty acid synthesis and regulation of carbohydrate sensors such as the factors controlling ChREBP. Another possible explanation for the lack of increased palmitate synthesis in response to insulin could be that this process is running at maximal capacity, with no room for further induction, due to the low fat diet. This is indicated in Figure 3A which shows that close to 100% of all palmitate in newly assembled TG molecules comes from the DNL pathway.

In contrast to the lack of change in *de novo* palmitate synthesis, significantly more adipose tissue accrual of labelled TG-glycerol was observed in the subcutaneous depot and significantly less lipolysis was observed in the gonadal depot. To exclude the possibility that this increase in TG-glycerol labelling could have been caused by insulin altering the exchange of deuterium from water into the glycerol moiety, we calculated the mean incorporation number (n) of hydrogens in TG-glycerol C-H bonds by combinatorial analysis (Mass Isotopomer Distribution Analysis), as described previously [10]. The value of n in TG-glycerol for each treatment group varied between 3.1 and 3.3 (data not shown) with no differences between treatment groups or depots. This indicates that the biosynthetic origin of glycerol was essentially entirely from glycolysis rather than glyceroneogenesis under these conditions of high dietary carbohydrate (for a thorough description of the theory and calculations see 10).

It is worth considering how insulin might cause adipose TG mass gain without changing the pool of fatty acids available from either *de novo* synthesis or diet. A few explanations are possible. Insulin may spare fatty acids from being used as fuel in beta-oxidation. Insulin reduces fatty acid oxidation in many tissues, including skeletal muscle [16], and also there have been other observations that insulin infusion causes increased body weight and/or adipose gain in the absence of a higher caloric intake [17–19]. Increased oxidation of protein in the whole animal to balance the reduced lipid oxidation, with redistribution of lean mass to adipose mass, seems unlikely here, as total body weight was increased in these animals by insulin treatment. Lean body mass weighs more per calorie (is less energy dense) than adipose tissue, so preferential oxidation of lean tissue would result in weight loss, which was not observed here. Measurement of food intake is relatively imprecise compared to metabolic measurements of adipose lipid content and metabolism, so modest effects on food intake might have been missed, as an alternate explanation. Indeed, Table 1 shows that both low and high insulin groups had non-significant trends toward higher food intake. Our findings indicate that insulin treatment likely reduced whole body fat oxidation rather than increasing *de novo* fatty acid synthesis, and altered TG deposition and lipolytic rates in different depots, but the whole-body macronutrient energetics responsible for insulin-induced increased gain in weight and adipose fat remain to be fully explained.

An intriguing finding in the current study was the differential response to insulin exhibited by the two distinct adipose depots. Of the 25±7mg TG gained pr day in excess compared to the control group in the subcutaneous depot from the HI group, 15±2mg originated from newly assembled TG, whereas only 2±4mg was newly deposited out of the 21±10mg TG accumulated pr day in the gonadal depot. Accordingly, these results suggest that if insulin indeed predisposes whole body metabolism towards less usage of energy in the form of fatty acid oxidation this would shunt these excess lipids towards the subcutaneous depot, whereas the gonadal depot instead responds by releasing less fatty acids. From the literature it is well known that these different types of depots differ in their interaction with insulin [20] and it emphasizes the importance of including visceral as well as non-visceral depots when investigating lipid dynamics.

## Conclusions

In the present study we found that insulin treatment of insulin sensitive, low-fat fed mice causes a significant gain of body weight and adipose mass within inducing measurable changes in either food intake nor DNL (as measured by *de novo* palmitate synthesis). These results point towards insulin predisposing fatty acids to altered storage efficiency (deposition and lipolysis) and perhaps beta-oxidation, rather than de novo synthesis. The two adipose depots studied also responded differentially to insulin treatment, with the subcutaneous depot responding by accumulating more newly deposited TGs whereas the gonadal depot preserved more TG by inhibiting lipolysis.
